# Influence of the jaw tracking technique on the dose calculation accuracy of small field VMAT plans

**DOI:** 10.1002/acm2.12029

**Published:** 2017-01-03

**Authors:** Ans C.C. Swinnen, Michel C. Öllers, Erik Roijen, Sebastiaan M. Nijsten, Frank Verhaegen

**Affiliations:** ^1^ Radiotherapy Department Maastro Clinic 6229 ET Maastricht The Netherlands

**Keywords:** calculation algorithm, dosimetry, jaw tracking technique, small fields

## Abstract

**Purpose:**

The aim of this study was to evaluate experimentally the accuracy of the dose calculation algorithm AcurosXB in small field highly modulated Volumetric Modulated Arc Therapy (VMAT).

**Method:**

The 1000SRS detector array inserted in the rotational Octavius 4D phantom (PTW) was used for 3D dose verification of VMAT treatments characterized by small to very small targets. Clinical treatment plans (*n* = 28) were recalculated on the phantom CT data set in the Eclipse TPS. All measurements were done on a Varian TrueBeamSTx, which can provide the jaw tracking technique (JTT). The effect of disabling the JTT, thereby fixing the jaws at static field size of 3 × 3 cm^2^ and applying the MLC to shape the smallest apertures, was investigated for static fields between 0.5 × 0.5−3 × 3 cm^2^ and for seven VMAT patients with small brain metastases. The dose calculation accuracy has been evaluated by comparing the measured and calculated dose outputs and dose distributions. The dosimetric agreement has been presented by a local gamma evaluation criterion of 2%/2 mm.

**Results:**

Regarding the clinical plans, the mean ± SD of the volumetric gamma evaluation scores considering the dose levels for evaluation of 10%, 50%, 80% and 95% are (96.0 ± 6.9)%, (95.2 ± 6.8)%, (86.7 ± 14.8)% and (56.3 ± 42.3)% respectively. For the smallest field VMAT treatments, discrepancies between calculated and measured doses up to 16% are obtained. The difference between the 1000SRS central chamber measurements compared to the calculated dose outputs for static fields 3 *×* 3, 2 *×* 2, 1 *×* 1 and 0.5 *×* 0.5 cm^2^ collimated with MLC whereby jaws are fixed at 3 *×* 3 cm^2^ and for static fields shaped with the collimator jaws only (MLC retracted), is on average respectively, 0.2%, 0.8%, 6.8%, 5.7% (6 MV) and 0.1%, 1.3%, 11.7%, 21.6% (10 MV). For the seven brain mets patients was found that the smaller the target volumes, the higher the improvement in agreement between measured and calculated doses after disabling the JTT.

**Conclusion:**

Fixing the jaws at 3 *×* 3 cm^2^ and using the MLC with high positional accuracy to shape the smallest apertures in contrast to the JTT is currently found to be the most accurate treatment technique.

## 1 Introduction

The use of small radiation fields in radiotherapy has increased substantially, in particular, in treatments with stereotactic beams and non‐uniform fields that are composed of multiple small subfields like in volumetric modulated arc therapy (VMAT), which has become the treatment of choice for an increasing number of treatment sites.^1^, ^2^, ^3^, ^4^, ^5^ This state‐of‐the‐art irradiation technique for the delivery of highly conformal radiation fields to the target volume requires complex dose calculation algorithms in the treatment planning system (TPS) as well as sophisticated medical linear accelerators (linacs). The rationale behind this increasing use of VMAT in stereotactic radiotherapy treatments is the possibility of improved healthy tissue sparing, the ability to create very steep dose gradients and the reduction in treatment time compared to intensity modulated radiation therapy (IMRT) and conventional treatments.^4^, ^5^

To exploit fully the benefits of stereotactic treatments, high spatial accuracy in dose delivery is vital. In stereotactic treatments, spatial and dosimetric accuracy are inextricably linked which makes the evaluation of the dosimetric accuracy of small field delivery equally important. The IPEM report no 103^6^ summarizes many studies discussing the difficulties of small field dosimetry and modeling. Accuracy could be limited due to the characteristics of the detectors used for measurements or due to approximations of Monte Carlo simulations used to model narrow beam doses in the TPS.^7^, ^8^

VMAT plans are typically highly modulated and a considerable fraction of the total control points used for a plan consists of small subfields.^9^ When evaluating VMAT plan accuracy, three aspects should be considered: the dosimetric measurements required for TPS commissioning, the beam model in the TPS, and the accuracy of verification measurements. The first and third aspect include the dosimetry of small fields, which is challenging due to the lack of charged particle equilibrium (CPE), partial blocking of the beam source giving rise to pronounced and overlapping penumbra and the availability of small detectors for sizes comparable to field dimensions.^10^ CPE is associated with the range of secondary particles and thus dependent on the beam energy and the density of the medium. The choice of radiation detectors in small fields is crucial as they usually perturb the secondary electron fluence due to its presence and composition. The second aspect influencing VMAT plan accuracy refers to properly designed multisource modeling using accurate measured data together with accurate dose calculation algorithms that handle non‐CPE conditions to provide acceptable dose distribution. It is important to realize the details of the improvements and limitations in the source model of the TPS and to know the inaccuracies of the input data which are used to optimize the source model parameters. The input data used for beam configuration which have an impact on the dose calculation accuracy are handled in section Method.B.

Several planning and measurement studies on clinical stereotactic VMAT plans for small target volumes have been published. For instance, Lagerwaard et al.^11^ and Verbakel et al.^12^ have carried out film measurements of VMAT plans for a range of tumor sizes, resulting in good agreement between the calculated and measured dose distributions. Remark that in these studies the convolution‐based anisotropic analytical algorithm (AAA) has been used, while this work evaluates the AcurosXB calculation algorithm which belongs to the class of the Linear Boltzman Transport Equation solvers, allowing – similarly to the Monte Carlo‐based methods – accurate modeling in heterogeneous media.^13^, ^14^ The accuracy of these two photon dose calculation algorithms (AAA, AcurosXB) compared to 2D and point measurements for small fields usable in stereotactic treatments has been investigated in Fogliata et al.^13^ and found to be acceptable using adequately tuned configuration parameters in the TPS, originally developed for standard external beam therapy for broad beams from conventional linear accelerators. Conversely, the work of Fog et al.^9^ cautioned about the use of small fields with VMAT and they reported large discrepancies up to 53% between measured and calculated doses for static fields one MLC leaf wide. For a small field VMAT plan with a 0.4 cm^3^ planning target volume about 10% overdosing was detected (Eclipse version 8.6, 2.5 mm grid spacing) and more modulation in the plan was measured than calculated.^9^

Since the investigation of Fog et al.^9^ has been performed using an older version of the TPS, the purpose of this work is to examine small field VMAT plan accuracy more thoroughly with a recent version of TPS and a new generation of linac.

Furthermore, the jaw tracking technique (JTT) provided by the latest types of linacs keeps jaws during dose delivery as close as possible to the MLC aperture, thereby minimizing leakage and transmission through the MLC leaves resulting in optimized organs‐at‐risk (OAR) sparing and potentially improving the dose falloff towards the surrounding critical structures.^15^ We aim to investigate the impact of disabling the JTT on the dose calculation accuracy to the target, for a range of small static fields as well as for a number of stereotactic radiosurgery treatments with very small target sizes. The suggestion to keep the jaw settings above a minimum size and to generate shielding and modulation by the MLC only, already appeared in the literature,^13^, ^16^ but to our knowledge has never been investigated thoroughly using clinical treatment plans for small treatment volumes.

## 2 Methods

To evaluate experimentally the accuracy of the dose calculation algorithm in the TPS in small field highly modulated VMAT treatments, 28 clinical VMAT treatment plans characterized by small target volumes and fraction doses between 2.75–30 Gy were delivered on a commercial high resolution 2D detector array in a rotational phantom for the independent 3D validation of the calculated dose distribution. Also, the influence of disabling the JTT on the dose calculation accuracy to the target is investigated for a range of small static fields as well as for a number of stereotactic radiosurgery treatments with very small target sizes. To validate the 2D detector array measurements, the small static fields were also delivered to radiochromic film (see Appendix B).

### 2.A Measurement system for 3D dose verification

The Octavius 4D system consists of an Octavius 4D phantom, a 2D detector array and a VeriSoft software package (version 6.2) for data collection and analysis (PTW‐Freiburg). Controlled by an inclinometer, the Octavius 4D phantom rotates synchronously with the gantry, taking time‐ and gantry angle‐resolved dose measurements.^17^ The detector array used in this work is the Octavius 1000SRS model which consists of 977 liquid‐filled ionization chambers covering an area of 11 × 11 cm^2^. Each detector covers a cross section of 2.3 × 2.3 mm^2^ with a height of 0.5 mm, resulting in an active volume of approximately 0.003 cm^3^. In the inner 5.5 × 5.5 cm^2^ area of the array, the centers of adjacent chambers are placed at a distance of 2.5 mm from each other.^18^ The detector size and the center‐to‐center distance of the detectors are important parameters for accurate spatial measurement of complex dose distributions with steep dose gradients. In Poppe et al.^18^ the good agreement of an unshielded Silicon diode and central chamber readings of the 1000SRS has been demonstrated for field sizes down to 1 × 1 cm^2^, proving the applicability of the array in small field dosimetry. In the same work, the consistency of 1000SRS measurement results and TPS calculations has been checked demonstrating that the detector array offers high spatial resolution in situations with rapidly changing dose gradients.

The beam incidence is always perpendicular to the detector array, avoiding the need of angular correction factors. The Octavius 4D algorithm is based on dose measurements at a certain depth in the phantom and on percentage depth dose (PDD) curves, independently measured with an ionization chamber in water at source‐to‐surface‐distance of 85 cm, that are used to reconstruct dose values along the ray lines that connect the relevant detectors and the focus of the beam.^17^ Prior to the measurement session, cross‐calibration of the Octavius 4D system was done by delivering a dose of 1 Gy using an open 5 × 5 cm^2^ field with no gantry rotation [Source Skin Distance (SSD) 84 cm, depth 16 cm, 6 MV and 10 MV photon beams]. Moreover, the same measurement set‐up was used to test the detector's measurement reproducibility, dose linearity and dose rate dependence (see Appendix A).

### 2.B Treatment planning

Treatment planning was carried out with the Eclipse TPS (Varian Medical Systems Inc., Palo Alto, CA, USA) version 11, using the AcurosXB photon dose calculation algorithm version 10.0.28. A calculation grid spacing of 2.5 mm is the default setting and is generally applied for clinical calculations as smaller grid spacing may require impractically large computation times. In addition, the minimum available grid spacing of 1 mm has been used for very small targets.

The Eclipse TPS was originally designed for 3D conventional broad photon beam radiotherapy treatments. Details of the most important incremental improvements from the point of view of accurate dose calculation for small field sizes are presented in Torsti et al.^16^ Currently, there are still limitations in the source model.^19^ It is important to remember that the head of the linac is not physically modeled. The complex interactions from all different components of the head can only be approximated: the phase space of the head scatter source is approximated as a planar distributed source with a Gaussian shape located at the bottom of the flattening filter. A second important source of scattered radiation in the head is located at the edge of the primary collimator. Furthermore, the source model parameters are optimized using symmetric jaw defined beam data with MLC retracted. It is a shortcoming that no asymmetric nor MLC‐delimited fields can be put into the configuration program. In our Eclipse Beam Configuration workspace, the imported beam data are measured relative profiles, depth dose curves and output factors (OF) for field sizes between 1 × 1 cm^2^ to 40 × 40 cm^2^ (respectively the smallest and largest possible field size fields as input, defined by jaws only) while the more concise set recommended by Varian contains field sizes defined by jaws between 3 × 3 cm^2^ and 40 × 40 cm^2^. As described in the Varian white paper,^16^ the relative profiles and depth dose curves below 3 × 3 cm^2^ can be imported in the configuration workspace but are not needed and smaller than 2 × 2 cm^2^ even not used in the configuration program, while the OF below 3 × 3 cm^2^ are used. How these OF for fields < 3 × 3 cm^2^ are applied in the TPS is discussed in section Conclusion. This means that the OF for small fields have to be measured very accurate, which is also stressed in the IPEM report no 103.^6^ An ideal detector for small field output factor measurements would be the one having a uniform spectral response, a high signal‐to‐noise ratio whilst being smaller than half the size of the region which can be considered acceptably uniform, and water equivalent. Unfortunately, there is no commercially available detector which can accurately measure OF of small radiation fields without requiring corrections for volume averaging or non‐water‐equivalent dosimetric properties. Therefore, it is ‘good practice’ to compare several different detector types.^6^ The use of a variety of detectors when measuring output factors for small fields helps to reduce the uncertainty in the estimation of the true value. In our case, we measured the OF between 1 × 1 cm^2^ and 3 × 3 cm^2^ with a range of detectors [IBA ionization chamber (10 mm^3^), IBA unshielded diode (0.75 mm^3^), PTW diamond detector (1 mm^3^)] with effective measuring point centered on the beam axis, by scanning the 50% dose which defines the field size, using the water tank software (Omnipro Accept; IBA, Schwarzenbruck, Germany) assuring a positioning accuracy < 0.7 mm. Multiple measurements are performed for each field size to determine the reproducibility of the measurements (which is overall within 0.5%). The small field OF from 3 × 3 cm^2^ down to 1 × 1 cm^2^ led to comparable measurement results for the investigated dosimeters (within 0.6% and 1.3% agreement for respectively 6 MV and 10 MV photons). In the Varian Beam Configuration Reference Guide is recommended that you do not need to measure all OF, just type in some values and interpolate the rest. However, we applied our own interpolation procedure using a selfmade matlab script leading to a less coarse result: for 1 × 1 cm^2^ the OF for 6 MV and 10 MV photon beams is 0.624 and 0.706 respectively, compared to the average measurement value of 0.650 and 0.712, which gives a deviation of 5% and 0.9%. From these findings, we estimate an inaccuracy on the OF measurements for 1 × 1 cm^2^ of maximum 5%.

The AcurosXB algorithm uses the multiple source model^19^ and was configured with the above mentioned set of input data. The modeling of the dynamic MLC deliveries is described through the use of two parameters related only to the energy, the leaf transmission (LT) and the dosimetric leaf gap (DLG) (which is the MLC gap assumed by Eclipse for closed MLC leaves to describe the transmission through the rounded MLC leaf tips). Our implemented values in the TPS are respectively LT = 1.2% and DLG = 0.11 cm for 6 MV and LT = 1.4% and DLG = 0.12 cm for 10 MV photon beams. In the configuration process, the focal spot size parameter, which models the physical effects of the finite size of the primary source (bremsstrahlung from target) by applying a Gaussian smoothing to the energy fluence of primary photons, is set to the default value of 1.0 mm for AcurosXB, corresponding to the recommendation in Fogliata et al.^13^ where values between 0.5 mm and 1 mm are suggested. The parameter affects the MU calculation for small fields and the penumbra region of the profiles for all field sizes.

Clinical patients (*n* = 28) were chosen with various lesion sites: lung, brain, prostate and spine. All were characterized by rather small to very small target volumes (PTV between 96.5 and 0.54 cm^3^) and fraction doses between 2.75–30 Gy. The clinical treatment plans were projected on the artificial homogeneous phantom CT data set provided with the Octavius 4D system and recalculated in the TPS. In the TPS, the electron density of the Octavius 4D phantom relative to water has been set to 1.016 according to the manufacturer's recommendation.

### 2.C Treatment technique

The treatment technique used was VMAT with RapidArc^®^ (RA) (Varian Medical Systems), which is based on an inverse planning method to deliver an intensity modulated dose distribution using a MLC of which the movement is modulated to the target volume while the beam is on and the gantry moves around the patient. This MLC movement can be very complex, containing many small field segments.

A TrueBeamSTx linac equipped with a High Definition (HD) 120‐MLC with inner leaf width of 2.5 mm (Varian Medical Systems, Palo Alto, USA) was used. This type of linac utilizes the JTT which keeps the collimator jaws during dose delivery by VMAT as close as possible to the MLC aperture, minimizing leakage and transmission through the MLC leaves.

### 2.D Treatment delivery

All data were gathered in 6 MV and 10 MV photon beams. By placing the 1000SRS array's active layer in the isocenter, the distances between the ionization chambers in the inner part of the array are thus equal to the projected leaf widths of the HD 120‐MLC at the isocenter. The effect of disabling jaw tracking, thereby fixing the collimator jaws at 3 × 3 cm^2^ and applying the MLC to shape the smallest apertures was investigated for static open squared fields of 3 × 3, 2 × 2, 1 × 1, 0.5 × 0.5 cm^2^ delivering the same amount of MU from the cross‐calibration field in an isocentric set‐up of Octavius 4D [i.e., SSD 84 cm, depth 16 cm, 5 × 5 cm^2^, 187 MU (6 MV), 152 MU (10 MV)]. To investigate in a static fields set‐up the differences in output for fields either created by the collimator jaws only (FS_jaws_) and by the MLC with jaws at a fixed 3 × 3cm^2^ position (FS_MLC_), a comparison between the 1000SRS central chambers measured dose output (Gy) and calculated by the TPS is made for both photon beam energies and for these two set‐up situations. Furthermore, the impact of JTT has been investigated for seven stereotactic patients with small brain metastases by comparing the measured and calculated dose distributions with JTT on the one hand and with collimator jaws fixed at 3 × 3 cm^2^ while the MLC is used to block the smaller parts on the other hand.

Prior to the measurements, the jaw position calibration has been verified using radiochromic EBT3 film (Ashland Inc., Covington, KY, USA) and the reproducibility of jaw position calibration with 0.1 mm can be confirmed by the regular quality control procedures following NCS report eight guidelines.^20^

### 2.E Octavius 4D analysis method

To evaluate the dosimetric agreement between the 1000SRS measured and calculated dose by Eclipse, a gamma evaluation method implemented in the Verisoft 6.2 software (PTW) was used^21^, ^22^ presenting the statistical results on the pass rates for different isodose levels as cut‐off dose value. We have selected 3D gamma criteria of 2% and 2 mm (local dose comparison) and cut‐off dose values of 10%, 50%, 80% and 95%. These values mean that voxels with doses below 10%, 50%, 80% and 95% are ignored in the gamma analysis and these were chosen to encompass most of the irradiated volume, focus on the higher dose regions and re‐evaluate the PTV coverage, respectively. Relative variation coefficients defined as (measured D_max_−TPS D_max_)/TPS D_max_ (%) give a comparison between the maximum dose values in the 3D distribution measured by the Octavius 4D system (measured D_max_) and calculated by the TPS (TPS D_max_).

### 2.F Verification of the impact of JTT using film

See Appendix B for the description of film measurements performed in this work.

## 3 Results

### 3.A VMAT treatment plan verifications

**Figure 1:**
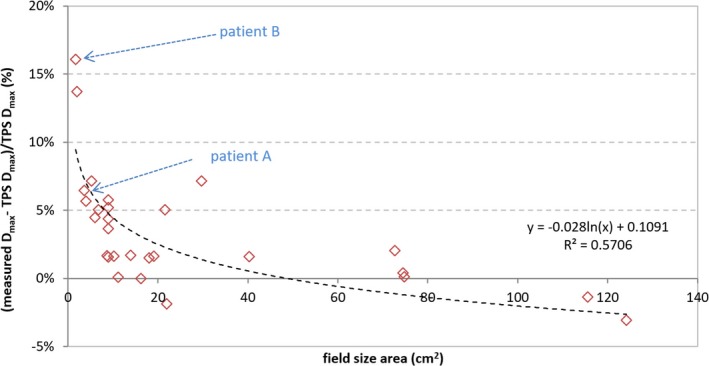
Plot of the relative differences in maximum dose as a function of field size area defined by collimator jaws for VMAT treatment plans. (D_max_ is referring to the maximum dose in the 3D volume; Patients A and B are further discussed in the section Results.A).

The errors in the device set‐up reproducibility were minimized by making cone beam computed tomography (CBCT) images of the set‐up before each set of measurements and using the online matching procedure for shifting the corresponding CBCT with the reference planning CT scan. Regarding the VMAT treatment fields, mean ± SD of the volumetric gamma evaluation scores for the dose difference and distance to agreement criteria of 2% and 2 mm with 10%, 50%, 80% and 95% cut‐off dose values were 96.0% ± 6.9%, 95.2% ± 6.8%, 86.7% ± 14.8% and 56.3% ± 42.3% (*n* = 28), respectively. An average pass rate of 96.0% with quite a large spread (6.9%) is found for the 10% cut‐off dose value. This may be caused by varying jaw settings in the different treatment plans. These jaw settings are the maximum jaw positions of the fields coming from the Arc Geometry tool in Eclipse, which suggests the geometry for the VMAT fields. Indeed, a trend can be observed for relative differences in the maximum dose as a function of the field size area of VMAT treatments going from very small to medium sized fields (Fig. 1). In general, the TPS underestimates the dose. The relative variation coefficients (measured D_max_−TPS D_max_)/TPS D_max_ (%) for the field size areas < 9 cm^2^ have deviations larger than 4.5% and even up to 16.1% for the smallest fields. For the dose calculations in this figure, a default calculation grid size of 2.5 mm has been used for field size areas > 9 cm^2^ and a calculation grid size of 1.0 mm for the field size areas ≤ 9 cm^2^.

The impact of using a calculation grid size of 1 mm instead of the default setting of 2.5 mm in treatment plans with very small fields was demonstrated for two of the 28 patients, for which large differences between measurements and calculations occur (indicated by patients A and B in Fig. 1). The relative variation coefficients (measured D_max_−TPS D_max_)/TPS D_max_ (%) show the effect of using a smaller grid size: 6.5% to 15% (patient A) and 16.1% to 21.2% (patient B) for respectively grid sizes 1 mm to 2.5 mm.

### 3.B Impact of disabling the JTT

#### 3.B.1 Octavius 4D system

Deviations between the central chamber measurements of the 1000SRS for open squared fields 3 × 3, 2 × 2, 1 × 1 and 0.5 × 0.5 cm^2^ collimated with the MLC whereby jaws are fixed at 3 × 3 cm^2^ and collimated with collimator jaws only are displayed in Table 1. The relative variation coefficients Δ_MLC_ = ((D_Oct4D MLC_−D_TPS MLC_)/D_TPS MLC_) (%) and Δ_jaws_ = ((D_Oct4D jaws_−D_TPS jaws_)/D_TPS jaws_)(%) describe the deviations between the measured and calculated doses for respectively static MLC defined beams (for field sizes smaller than 3 × 3 cm^2^ the jaws are fixed at this position) and the beams collimated by the jaws. The data belonging to FS_MLC_ in contrast to FS_jaws_ (Table 1) are not affected by the OF down to 1 × 1 cm^2^ used for beam configuration. For field sizes < 2 × 2 cm^2^, the quantity |Δ_(Oct4D,TPS)jaws_−Δ_(Oct4D,TPS)MLC_|(%) (Table 1) represents an improvement in dose calculation accuracy that can be achieved by fixing the jaws at 3 × 3 cm^2^ and letting the MLC form the smallest apertures.

**Table 1:** Static fields collimated with MLC versus with collimator jaws only for 6 MV (white rows) and 10 MV (shaded rows)

FS_MLC_ (cm^2^)	D_Oct4D MLC_ (Gy)	D_TPS MLC_ (Gy)	Δ_(Oct4D, TPS) MLC_ (%)	FS_jaws_ (cm^2^)	D_Oct4D jaws_ (Gy)	D_TPS jaws_ (Gy)	Δ_(Oct4D, TPS) jaws_ (%)	|Δ_(Oct4D,TPS)jaws_−Δ_(Oct4D,TPS)MLC_|(%)
3 × 3	0.924	0.930	−0.6%	3 × 3	0.926	0.930	−0.4%	0.2%
2 × 2	0.891	0.895	−0.4%	2 × 2	0.885	0.882	0.3%	0.8%
1 × 1	0.813	0.806	0.9%	1 × 1	0.786	0.730	7.7%	6.8%
0.5 × 0.5	0.653	0.628	4.0%	0.5 × 0.5	0.568	0.518	9.7%	5.7%
3 × 3	0.934	0.933	0.1%	3 × 3	0.935	0.933	0.2%	0.1%
2 × 2	0.890	0.879	1.3%	2 × 2	0.881	0.859	2.6%	1.3%
1 × 1	0.761	0.738	3.1%	1 × 1	0.721	0.628	14.8%	11.7%
0.5 × 0.5	0.577	0.535	7.9%	0.5 × 0.5	0.519	0.401	29.4%	21.6%

FS_MLC_, field size collimated by MLC with jaws fixed at 3 × 3 cm^2^; FS_jaws_, field size collimated by collimator jaws; D_TPS MLC_, calculated dose output of static fields collimated by MLC; D_Oct4D MLC_, dose output of static fields by MLC measured with Octavius 4D system; D_TPS jaws_, calculated dose output of static fields collimated by jaws; D_Oct4D jaws_, dose output of static fields by jaws measured with Octavius 4D system; Δ_(Oct4D,TPS)MLC_(%) = ((D_Oct4D MLC_−D_TPS MLC_)/D_TPS MLC_) (%); Δ_(Oct4D,TPS)jaws_(%) = ((D_Oct4D jaws_−D_TPS jaws_)/D_TPS jaws_).

The gamma agreement scores and comparison of measured versus calculated maximum doses for seven clinical patients with small brain lesions are shown in Table 2. The patients are ordered in such a way that the smallest field size area belongs to patient 1 and the largest to patient 7. Re‐planning of the seven patients with disabling of the JTT and fixing the collimator jaws at 3 × 3 cm^2^ leads to a substantial improvement in the agreement scores for the higher dose regions in Table 2. From Table 2 can also be seen that there is an improvement in correspondence between measured and calculated maximum doses after fixing the collimator jaws at 3 × 3 cm^2^. In general, it can be noticed that the improvement is increasing with decreasing field size.

**Table 2:** Measured versus calculated maximum doses with jaw tracking on (white rows) against fixed collimator jaws at 3 × 3 cm^2^ (shaded rows) for seven brain metastases patients

Patients	PTV (cm^3^)	10%	2%/2 mm cut‐off			TPS D_max_ (Gy)	Measured D_max_ (Gy)	(Measured D_max_−TPS D_max_)/TPS D_max_(%)	Field size (cm^2^)
			50%	80%	95%				
1	0.61	69.5%	75.2%	7.4%	0.0%	7.81	9.06	16.1	1.4 × 1.2
		99.9%	97.7%	89.3%	0.0%	8.77	9.27	5.8	3.0 × 3.0
2	0.54	80.6%	84.0%	34.6%	0.0%	23.40	26.61	13.7	1.4 × 1.5
		92.6%	96.4%	84.6%	20.0%	25.38	26.70	5.2	3.0 × 3.0
3	0.74	99.8%	94.6%	81.0%	16.7%	8.55	8.93	4.5	1.6 × 1.4
		99.9%	97.0%	89.8%	50.0%	8.67	8.97	3.5	3.0 × 3.0
4	0.79	91.1%	94.0%	76.2%	0.0%	7.69	8.18	6.5	1.9 × 1.9
		100.0%	98.8%	95.3%	71.4%	7.97	8.26	3.6	3.0 × 3.0
5	1.11	99.6%	90.6%	68.9%	6.7%	7.18	7.59	5.7	2.0 × 2.0
		99.8%	95.0%	82.6%	25.0%	7.50	7.78	3.7	3.0 × 3.0
6	1.32	96.7%	94.2%	70.0%	0.0%	9.57	10.26	7.2	2.4 × 2.2
		98.0%	96.9%	83.9%	28.6%	9.75	10.18	4.4	3.0 × 3.0
7	2.43	99.8%	100.0%	100.0%	100.0%	8.55	8.70	1.7	3.0 × 2.9
		99.9%	100.0%	100.0%	100.0%	8.57	8.70	1.6	3.0 × 3.0

D_max_, maximum dose; the 3D gamma criteria of 2% and 2 mm (local dose) present the statistical results on the pass rates for different isodose levels between 10% and 95% as cut‐off dose value.

#### 3.B.2 EBT3 film

The results on the comparison between EBT3 film and Octavius 4D measurements can be found in Appendix B.

## 4 Discussion

In this work, the main purpose was to quantify the accuracy of the dose calculation algorithm for clinical small field VMAT plans.

The user of the TPS has little influence on the number of subfields to be included in the plan – as there is no parameter which controls the smoothness of dose modulation – nor has the user information on the smallest field size for which dose computation has acceptable accuracy. The only ability the user has to minimize the errors arising from unnecessary use of very small subfields in RA plans is to use a MU constraint. As a rule of thumb, we accept VMAT treatment plans with a maximum number of MU equal to 3 times the prescription dose (in cGy). 62% of the VMAT plans in this study have 10% or more of their fields smaller than 2 cm^2^. So 62% of the VMAT plans are directly affected by the uncertainties in small field dosimetry. The Octavius 4D measurements of the VMAT treatment fields suggest that the use of very small subfields in small field VMAT plans may be the cause of the high discrepancies between the calculated and measured doses (Fig. 1).

A possible explanation to the trend observed in Fig. 1 can be that Eclipse was originally designed for 3D conformal radiotherapy and that – even after several important and effective improvements – there are still limitations in the Eclipse source model for small fields, which were mentioned in section Method.B.

**Figure 2:**
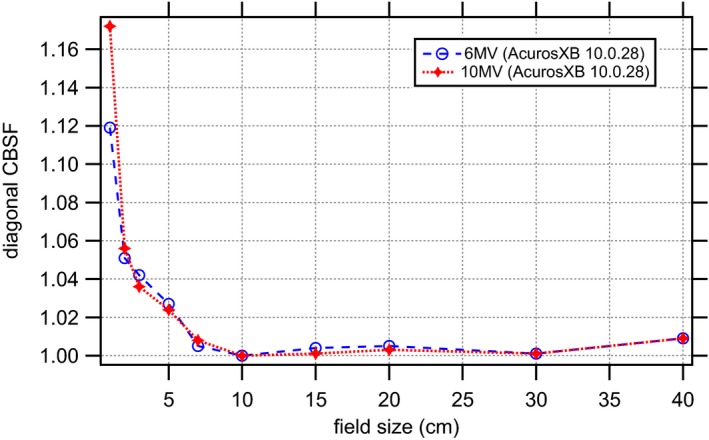
Calculated diagonal CBSF for symmetric and square jaw defined fields for a TrueBeam STx with 6 MV and 10 MV photon beams.

Furthermore, if we indulge in how the OF are handled in Eclipse, we have to consider the collimator backscatter factor (CBSF) table (Fig. 2), calculated at the last stage of the beam configuration process. Once the beam model has been created from the profiles and depth dose curves, the configuration program basically calculates a series of rectangular and square fields on a water phantom using the current configuration, and is compared to the equivalent values from the user inputted output factor table. The CBSF is essentially the ratio of calculated point dose versus measured output factor [formula (1)].^23^ When performing an AcurosXB dose calculation, the CBSF table is used as a look‐up table to correct the monitor units for the required dose. The final MU are calculated from the prescribed dose, plan normalization, field weight, field normalization and a normalization factor determined by the dose calculation algorithm. The normalization factor determined by AcurosXB is the MU value for 1 Gy to 100% of the current field. AcurosXB calculates the monitor units at the normalization point MU_norm_ for open or wedged fields [formula (2)]:^23^

(1) $$CBSF\left(X,Y\right)=\frac{OF_{ref}}{{\underbrace{OF(X,Y)}}_{measured}}\cdot \frac{D^{\prime }\left(X,Y\right)}{{\underbrace{D_{ref}^{\prime }}}_{TPS}}$$

(2) $$MU_{norm}=CBSF\left(X,Y\right)\cdot \frac{MU_{calib}}{{\underbrace{D_{calib}}}_{measured}}\cdot \frac{D_{ref}}{{\underbrace{D_{norm}\left(X,Y\right)}}_{TPS}}\cdot \frac{1}{{\underbrace{WCF}}_{TPS}}$$

With CBSF(X, Y) being the CBSF for the collimator settings in X and Y direction. OF_ref_ is the output for the reference conditions (10 × 10 cm^2^, 5 cm depth, SSD 100 cm and 100 MU) and is equal to 1. OF(X,Y) is the output factor for the collimator settings in X and Y direction. D′(X,Y) is the dose at the reference point as calculated by the dose calculation algorithm for collimator setting X,Y. D′_ref_ is the dose at the reference point for the reference conditions mentioned above.

MU_calib_ and D_calib_ are the number of monitor units and dose under calibration conditions (10 × 10 cm^2^, d_max_, SSD 100 cm. 100 MU correspond to 1 Gy).

D_ref_ is the dose for the reference conditions at the calibration depth (d_max_).

D_norm_(X,Y) is the dose calculated by the TPS at the chosen normalization point for collimator setting X and Y.

WCF is a wedge correction factor for hard field wedges and not applicable in our case (WCF = 1).

Obviously, the observed CBSF table shape (Fig. 2) looks quite unphysical compared to the measured physical collimator backscatter factors at small and large field sizes. Of course we would expect the CBSF to be highest for the smallest field size, namely at 1 × 1 cm^2^, but this “jump” in the curve cannot be due to inaccuracies in the measurement of output factor at 1 × 1 cm^2^ alone. The differences between the physical back scatter factors and the configured values reveal information about the remaining limitations of the source model and are due to the fact that the CBSF in the source model beam data is a residual correction factor taking into account all phenomena of phantom scatter and head scatter that are not otherwise accounted for by the source model or the dose deposition engine.^16^

Finally, a shortcoming in the Eclipse TPS version 11 is the fact that TrueBeam cannot be selected as Machine Type in the Parameter View of the Beam Configuration program. Varian advises the customers to use a C‐series instead although some difference in the backscatter factors between C‐Series and TrueBeam machines can be expected.^24^ Recently published papers about CBSF show a large difference between the CBSF in Torsti et al.^16^ and the ones published by Sibolt et al.^25^ and Zavgorodni et al.^24^ Sibolt et al.^25^ discusses the increased shielding of the monitor chamber in the treatment head in TrueBeam linacs of with respect to Clinacs. Moreover, linac head geometry is not made available for TB linacs, which makes it impossible to explicitly model the backscatter to the monitor chamber.

When configuring the beam data in Eclipse, symmetrical open squared fields are used in the process: only PDD and profiles for field sizes of 3 × 3 cm^2^ up to 40 × 40 cm^2^. The dosimetric data for the smaller fields can be imported but are not used by the system for beam modeling. Currently, no asymmetrical field sizes nor MLC shaped fields are used in Eclipse when configuring the beam data. Output factors for fields smaller than 3 × 3 cm^2^_,_ on the other hand, should be measured very accurately with a reliable detector, since these are applied in the dose calculation. Up to now, Varian does not recommend the use of output factors for very small fields in the configuration process.^13^ Differences up to 5% between measurements and calculations of small field output for 6 MV photon beams were found for configurations including the 3 × 3 cm^2^ as minimum field size,^13^ which is of the same order what we found in Δ_(Oct4D,TPS)MLC_(%) for 6 MV photons in Table 1. For 10 MV photon beams, this factor in Table 1 shows even larger differences. We found that fixed jaws for field sizes smaller than 3 × 3 cm^2^ (FS_MLC_ in Table 1) leads to better agreement between measured and calculated maximum doses, as these data are not affected by the OF down to 1 × 1 cm^2^ used for beam configuration. Table 2 illustrates that the smaller the field size, the larger the impact of jaw fixation at 3 × 3 cm^2^ on the dose difference results: the improvement in agreement between measured and calculated doses is the highest for the smallest target volume. Therefore, as already suggested in the literature^13^, ^16^ but never reported for clinical treatment plans, disabling jaw tracking is an option to increase the dose prediction accuracy by Eclipse for very small target volumes. Moving the jaws away from the leaf ends, typically 1 to 2 cm, increases the output and penumbra width for small fields because of more photons leaking through the leafs.^26^ From Table 2 we believe that the TPS models this jaw effect on the small MLC fields quite well. Retracting the jaws to 3 × 3 cm^2^ can lead to higher OAR doses, but AcurosXB predicts the dose under small MLC defined field segments well.^27^ Focusing on the gamma agreement scores for the lower cut‐off dose regions in Table 2, it can be seen that the MLC leakage is well modeled by the TPS. Moreover, out‐of‐field doses in regions shielded by the MLC (or both the MLC and the jaws) in RA plans were studied in Fogliata et al.^28^ demonstrating the good agreement between measurements and calculations with AcurosXB. Regarding the higher dose to the OAR, Feng et al.^15^ showed that both jaw tracking and static jaw IMRT plans can achieve comparable target dose coverage, and that OAR sparing with JTT is of more clinical importance for the patients with large and complex targets close to highly radio‐sensitive organs.

**Figure 3:**
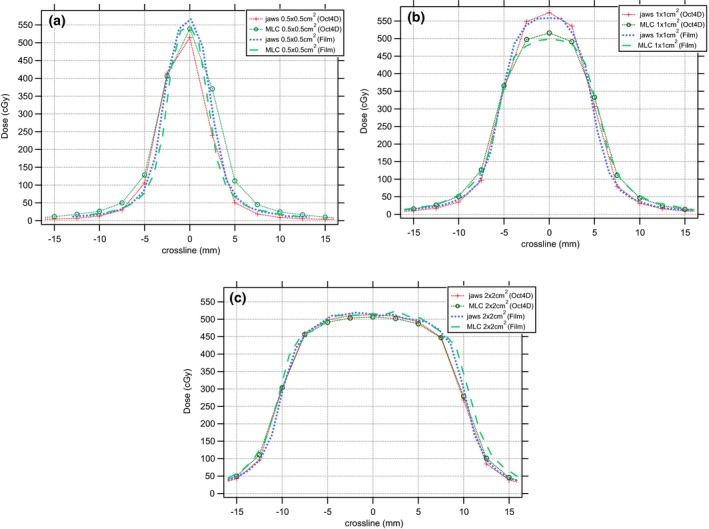
Comparison of EBT3 (film) and Octavius 1000SRS (Oct4D) for static 10 MV beams collimated with either MLC or jaws with field sizes (a) 0.5 × 0.5 cm^2^, (b) 1 × 1 cm^2^, (c) 2 × 2 cm^2^.

To validate the measurements done with the Octavius 4D system concerning the improvement of fixing the jaws, we compared the 1000SRS results for static fields with EBT3 film (see Appendix B). Although it is a 2D detector, radiochromic films offer many unique features. Besides weak energy dependence, it provides sub‐millimeter spatial resolution to compare with our 1000SRS measurements. Figs. 3(b) and 3(c) show a good agreement between both detector methods. For both film and 1000SRS, the same magnitude of improvements, represented by |Δ_(Oct4D,TPS)jaws_−Δ_(Oct4D,TPS)MLC_|(%) in Table 1 and |Δ_(Oct4D,EBT3)jaws_−Δ_(Oct4D,EBT3)MLC_|(%) in Table 3, can be achieved by fixing the jaws at 3 × 3 cm^2^ for the field sizes smaller than 3 × 3 cm^2^ and letting the MLC shape the smallest apertures. However, the 1000SRS measurements for the 0.5 × 0.5 cm^2^ fields (Fig. 3(a)) show less deviation with TPS than the ones by film, which can be explained by the fact that this field size is probably too small for correct measurement by the 1000SRS. In this situation, the focal spot (source) will be almost fully occluded by the collimator jaws or MLC as seen from the isocenter. For the latter 2D comparison, the spatial resolution of EBT3 film is superior to the 1000SRS.

**Table 3:** Comparison of the dose outputs for static fields collimated with MLC and with jaws using Octavius 4D and EBT3 film

FS_MLC_ (cm^2^)	D_EBT3 MLC_ (cGy)	D_Oct4D MLC_ (cGy)	Δ_(Oct4D,EBT3)MLC_(%)	FS_jaws_ (cm^2^)	D_EBT3 jaws_ (cGy)	D_Oct4D jaws_ (cGy)	Δ_(Oct4D,EBT3)jaws_(%)	|Δ_(Oct4D,EBT3)jaws_−Δ_(Oct4D,EBT3)MLC_|(%)
2 × 2	515.8	506.5	−1.8%	2 × 2	512.9	513.0	0.0%	1.8
1 × 1	499.4	515.0	3.1%	1 × 1	558.1	574.0	2.9%	0.2
0.5 × 0.5	547.0	539.0	−1.5%	0.5 × 0.5	566.6	515.0	−9.1%	7.6

FS_MLC_, field size collimated by MLC with jaws fixed at 3 × 3 cm^2^; FS_jaws_, field size collimated by collimator jaws; D_EBT3 MLC_, measured dose output by film of static fields collimated by MLC; D_Oct4D MLC_, dose output of static fields by MLC measured with Octavius 4D system; D_EBT3 jaws_, measured dose output by film of static fields collimated by jaws; D_Oct4D jaws_, dose output of static fields by jaws measured with Octavius 4D system; Δ_(Oct4D,EBT3)MLC_(%) = ((D_Oct4D MLC_−D_EBT3 MLC_)/D_EBT3 MLC_) (%); Δ_(Oct4D,EBT3)jaws_ (%) = ((D_Oct4D jaws_−D_EBT3 jaws_)/D_EBT3 jaws_) (%).

## 5 Conclusion

Doses calculated for stereotactic VMAT plans show an acceptable agreement against measurements with the 1000SRS in the Octavius 4D system for field sizes > 2 × 2 cm^2^. For very small highly modulated VMAT fields larger discrepancies are obtained demonstrating the need for improvement in dose calculation for small fields. Fixing the jaws at the minimum required field size for beam data input in the TPS, i.e., 3 × 3 cm^2^, and using the MLC with high positional accuracy to shape the smallest apertures in contrast to jaw tracking is currently found to be the most accurate treatment technique.

To conclude, our recommendations to minimize the effects of calculation uncertainties in RA plans for very small target sizes:

- A calculation grid size of 1 mm is found to be superior to 2.5 mm.
- An upper MU constraint should be assigned during optimization to avoid plans with high modulation and very high number of MUs increasing treatment time.
- Fixed collimator jaws at 3 × 3 cm^2^ should be used for small target volumes where the JTT leads to jaw defined field sizes smaller than 3 × 3 cm^2^.

## Conflict of Interest

The authors declare no conflict of interest.

## Appendix A

### A.1

Even without pre‐irradiation, the response of the 1000SRS detector array in the Octavius 4D phantom (SSD 84 cm, depth 16 cm) shows good reproducibility (coefficient of variation within 0.5% in the dose range of 0.01 to 7 Gy). A near‐perfect linear relationship between delivered dose and 1000SRS reading was confirmed in the same dose range [monitor units (MU) between 1–1000] for both photon energies. These measurements confirm the findings in Poppe et al.^18^, where excellent linearity has been demonstrated up to 20 Gy.

Due to the low drift velocities in liquids, recombination losses in higher dose rates should be investigated. The dose rate dependence has been determined by delivering a constant number of 100 MU for dose rates between 10 and 2400 MU/min (dose rates higher than 600 MU/min are coming from Flattening Filter Free beams), resulting in a mean deviation of the CAX dose readings (normalized to the nominal dose rate) of 0.3% for all photon beam energies.

## Appendix B

### B.1

To check further the consistency of 1000SRS measurement results and TPS calculations, the static open squared fields 2 × 2, 1 × 1, 0.5 × 0.5 cm^2^ were also irradiated on radiochromic EBT3 film batch n^o^ 04151402 (Ashland Inc., USA) placed at a measurement depth of 16 cm (SSD 84 cm) inside a water‐equivalent solid RW3 phantom (1.045 g/cm^3^) in the form of 40 × 40 cm^2^ plates of 1 cm thickness (PTW‐Freiburg), in accordance to the Octavius 4D set‐up but with a dose of 5 Gy and beam energy of 10 MV.

In addition to film irradiation, we performed ionization chamber measurements with a cross‐calibrated PTW Farmer chamber (type TN30012, SN 0158) at the depth of 5 cm (SSD 100 cm, 10 × 10 cm^2^, 5 Gy, 10 MV). One film in this set‐up was used for calibration. Also, a blank film from the same batch was applied as background.

The films were scanned under a 4 mm glass plate with a flatbed A3‐size Epson 11000XL scanner with built‐in transparency unit and analyzed using the one‐scan protocol by Lewis et al.^29^ by means of the FilmQA Pro software (version 5.0.5470.35091), a quantitative analysis tool designed for film scanning, pixel value conversion into dose and comprehensive dose analysis (Ashland Inc., USA).

Further recommendations mentioned in Mathot et al.^30^ were followed. Due to the anisotropic light scattering in radiochromic films, film orientation must be kept constant, which is in this study “landscape” (when the long axis of the film is perpendicular to the scanner lamp). Furthermore, the reproducibility of film positioning is an important issue due to the non‐uniform scanner response over the scan field perpendicular to the scan direction. The well‐known “lateral response effect” causes transmission pixel values to decrease as the lateral distance from the scan axis increases. Therefore, we used a ruler template on the scanner glass so that film pieces can be placed in the central scan axis of the scanner bed. Due to the small field size and accurate positioning of the film in the center of the scanner, lateral response artefacts, turned out to be negligible.^31^

The comparison between the EBT3 film with Octavius 4D measurements for open squared fields 2 × 2, 1 × 1 and 0.5 × 0.5 cm^2^ collimated with the MLC (whereby jaws are fixed at 3 × 3 cm^2^) and collimated with collimator jaws only are displayed in Figs. 3(a)–3(c). The central axis absolute dose outputs and the relative variation coefficients Δ_(Oct4D,EBT3)MLC_ = ((D_Oct4D MLC_−D_EBT3 MLC_)/D_EBT3 MLC_)(%) and Δ_(Oct4D,EBT3)jaws_ = ((D_Oct4D jaws_−D_EBT3 jaws_)/D_EBT3 jaws_)(%) can be found in Table 3. Factors Δ_(Oct4D,EBT3)jaws_ and Δ_(Oct4D,EBT3)MLC_(%) in Table 3 and Figs. 3(b) and 3(c) show a good agreement between both detector methods, except for 0.5 × 0.5 cm^2^ fields (Fig. 3(a)), where the resolution of the 1000SRS is limiting.
